# Usefulness of Intraoperative Neurophysiological Monitoring in Intradural Spinal Tumor Surgeries

**DOI:** 10.3390/jcm13247588

**Published:** 2024-12-13

**Authors:** Lidia Cabañes-Martínez, Olga Fedirchyk-Tymchuk, Laura López Viñas, Federico Abreu-Calderón, Rodrigo Carrasco Moro, Marta Del Álamo, Ignacio Regidor

**Affiliations:** 1 Clinical Neurophysiology Department, Hospital Universitario Ramón y Cajal, 28034 Madrid, Spain; lidia.cabanes@salud.madrid.org (L.C.-M.);; 2 Clinical Neurophysiology Department, Hospital Regional Universitario de Málaga, 29010 Málaga, Spain; 3 Neurosurgery Department, Hospital Universitario de Torrejón, 28850 Madrid, Spain; 4 Neurosurgery Department, Hospital Universitario Ramón y Cajal, 28034 Madrid, Spain

**Keywords:** spinal cord tumors, intraoperative neurophysiological monitoring, somatosensory-evoked potentials, SSEP, motor-evoked potentials, MEP, D-wave, outcome

## Abstract

**Objective:** Due to the absence of studies supporting the role of intraoperative neurophysiological monitoring (IONM) in intradural spinal tumors, this study evaluates the clinical outcome after these surgeries in relation to the use of the advanced intraoperative neurophysiological techniques. **Methods:** This is an observational, descriptive and retrospective study of two cohort groups in relation to the presence or absence of IONM during the intervention and the subsequent evaluation of the clinical and functional results in the short and medium terms. Ninety-six patients with extra- or intramedullary intradural spinal tumors operated on by the neurosurgery team of our center completed the current study. **Results:** We observed improvements in the Prolo, Brice and McKissock and McCormick scales scores in the monitored patients. These results examine the usefulness of IONM to preserve neurological functions and, therefore, its impact on quality of life. The rate of neurological deficits in the unmonitored patients was 14.5%, whereas it was 8.3% of the patients whose treatment included IONM. **Conclusions:** It is important to emphasize the importance of implementing IONM for early recognition of possible neurological damage, the improvement of postoperative functional outcomes, and for decreasing the rate of neurological complications. **Significance:** This study provides reliable results on the importance of IONM in intradural spinal tumor surgeries.

## 1. Introduction

Primary intradural spinal tumors constitute about 2–4% of central nervous system tumors [1,2]. Intradural extramedullary spinal cord tumors constitute approximately two-thirds of spinal tumors, and the other one-third are intramedullary spinal cord tumors [3]. The approach to surgical management has changed significantly since the onset of the use of microsurgical techniques and intraoperative neurophysiological monitoring (IONM) [4,5]. In analogy to the introduction of the use of the microscope, the use of IONM was initially not widely accepted by all neurosurgeons. But, since then, IONM has become one of the fastest-developing fields in modern neurosurgery, especially in the last three decades. It is known that the implementation of its techniques in the operating room is not new; however, the possible applications of IONM have expanded and evolved in an important way. The development of recording motor-evoked potentials under general anesthesia has also greatly enhanced the role of the IONM and has also guided the progress of clinical research, particularly in spinal cord surgery [5]. Consequently, intraoperative neurophysiology can play an important role in the decision making by predicting potential neurological injuries that may arise or worsen during the procedure. This is supported by the fact that the decision to indicate surgical treatment for these tumors depends also on anatomical or imaging factors and not only on oncological factors (expected survival, malignancy, or tumor extent).

In recent years, the use of IONM has allowed surgeons to modify their procedures according to the neurophysiological findings obtained during the surgery, as well as to advise about postoperative prognosis. However, because any randomly controlled study to evaluate the effectiveness of IONM is restricted by the ethical problems secondary to the randomization of its use, no meta-analytic studies to support the advantages of resecting tumors with IONM have been conducted. Given this dilemma, the present study is an observational, descriptive, and retrospective study examining the advantages of IONM, both during the surgery and for the establishment of a post-surgical prognosis.

## 2. Materials and Methods

**Figure 1:**
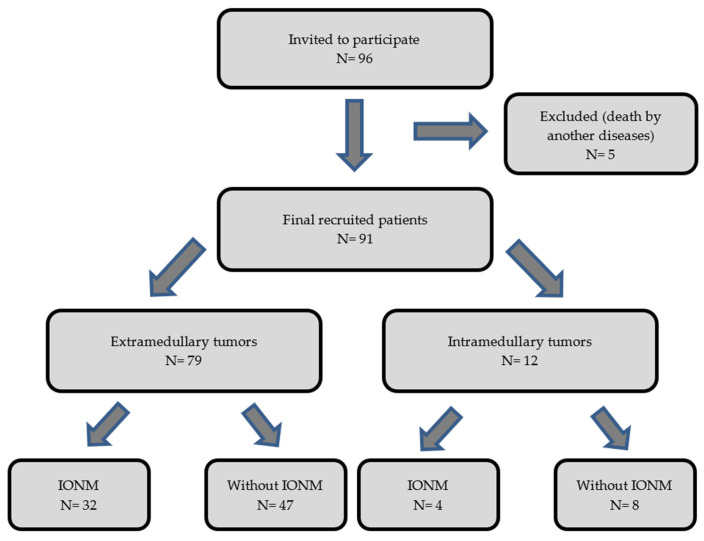
Diagram showing the flow of the patients through the study.

The purpose of our work has been to objectively analyze and assess the benefit of the use of IONM in intradural tumor surgeries to determine its impact on the postoperative prognosis of the operated patients. We performed an observational, descriptive, and retrospective cohort analysis of two groups of patients (operated with IONM and without IONM) during the intervention. We collected data from 96 patients with an intradural spinal tumor who were surgically treated from January 2002 to December 2013 by a single neurosurgery team at our center. The inclusion criteria for the study were age of majority (>18 years of age) and diagnosis of primary intradural spinal tumor (extra- or intramedullary). We excluded patients diagnosed with spinal metastases and those who underwent re-interventions due to recurrence of the tumor. Of the 96 eligible patients, 5 were excluded from the sample due to exitus in the early postoperative period caused by complications unrelated to the surgery (two patients with PNET; one with disseminated primary diffuse leptomeningeal gliomatosis; one case of intradural meningioma who had a massive subarachnoid hemorrhage 3 days after the surgery; and one case of intramedullary glyoblastoma who acquired a nosocomial bilateral pneumonia, which caused death). Of the remaining 91 patients, 79 had extramedullary tumors and 12 had intramedullary tumors. Within the group with an extramedullary tumor location, 32 patients were monitored and 47 were not monitored. Among the patients with intramedullary tumors, 4 were monitored and 8 were not monitored (Figure 1). We show all the data collected in Table 1 and Table 2.

The decision to request IONM depended on the personal criteria of each one of the neurosurgeons, mostly influenced by the previous experience with IONM. Younger surgeons requested this technique more frequently. Other factors determining the use of IONM were the location and type of the tumor and the clinical situation of the patient. There might be other reasons that we are not aware of contributing to the decision of the responsible neurosurgeons. Given that a randomization was not possible in this study due to an obvious ethical conflict, we used this arbitrariness in the decision on when to monitor as a way of “pseudo-randomization”. Clinical evaluations were conducted 2–3 months after surgery.

Our study was approved by the Clinical Research Ethics Committee of Hospital Universitario Ramón y Cajal (code n°187-16), according to the national legislation established by the time of the study (Royal Decree 1090/2015 and CAM Decree 39/1994 of the Community of Madrid).

### 2.1. Intraoperative Neurophysiological Monitoring (IONM)

Informed consent for IONM was obtained with informed consent for the surgery. IONM was performed by a senior consultant specialized in IONM. A standard protocol of neurophysiological techniques, with some modifications based on injury level, was employed.

#### 2.1.1. Motor Pathway Monitoring: Motor-Evoked Potentials (MEPs) Elicited via Transcranial Electrical Stimulation (tES)

- Muscle motor-evoked potentials (mMEPs): Stimulation was performed using ‘corkscrew’ electrodes at C3–C4 (according to the international 10/20 electrode placement system). A stimulus protocol consisted of 5-pulse trains (pulse duration of 0.5 ms and interstimulus interval (ISI) of 2 ms) and a maximum current intensity of 200 mA, according to the technical specifications of the equipment. Recording parameters were a 500 Hz low-pass filter and a 5 kHz high-pass filter, with recordings taken from the intercostal muscles (T3–T5), anterior rectus abdominis, vastus lateralis quadriceps, anterior tibialis, and both abductor hallucis and abductor pollicis brevis using paired monopolar needles.
- Epidural motor-evoked potentials (D-wave): Single 0.2 ms pulses were delivered using ‘corkscrew’ electrodes placed at C3–C4. Recordings were obtained using two epidural electrodes, positioned cranially and caudally relative to the surgical site, with a low-pass filter set to 20 Hz and a high-pass filter set to 2 kHz.

#### 2.1.2. Sensory Pathway Monitoring: Somatosensory-Evoked Potentials (SSEPs)

- Lower-limb SSEPs: elicited by stimulating the posterior tibial nerve at the internal malleolus using repetitive electrical pulses (0.2 ms duration, trigger rate of 4.690 Hz).
- Recordings were performed in the popliteal fossa with monopolar needle electrodes and on the scalp at the Cz’-Fz lead using ‘corkscrew’ electrodes. Data were processed with a low-pass filter set to 20 Hz and a high-pass filter set to 2 kHz, averaging 200 sweeps.
- Upper-limb SSEPs: elicited by stimulating the median nerve at the wrist using similar parameters as above, with recordings from the Erb’s point and the scalp at the Cc’-Fz lead. An additional recording channel for spinal cord somatosensory responses with epidural electrodes was used in some cases.

#### 2.1.3. Specific Monitoring Techniques for Tumors Located in Lower Spinal Segments

- Bulbocavernous reflex (BCR): elicited using a stimulation protocol consisting of 5-pulse trains (0.5 ms pulse duration and ISI of 2 ms) of the dorsal nerve of the penis (in male patients) or dorsal nerve of the clitoris (in female patients). Reflex responses were recorded from the external anal sphincter bilaterally using monopolar needles (bulboanal reflex).
- Motor root mapping: we used a monopolar stimulator delivering 0.1 ms cathodal pulses at 1 Hz frequency, referred to a distant anode (a needle placed near the surgical site). Recordings were obtained from the quadriceps, anterior tibialis, gastrocnemius, abductor hallucis, and bilateral external anal sphincter muscles.
- Free-run EMG: responses were recorded from the muscle groups mentioned above, with a low-pass filter set to 20 Hz and a high-pass filter to 10 kHz.

### 2.2. Clinical-Functional Evaluation

Clinical outcomes were assessed using the Prolo scale [6,7], Brice and McKissock scale [8,9], and McCormick scale [10,11]. In addition, the presence of neurological deficits that either appeared or were aggravated after the intervention was evaluated. The Prolo scale (Table 3) evaluates functional states affecting patient autonomy, considering pain severity, neurological function, economic impact on daily life, and medication use on a scale from 1 to 4, with higher scores indicating better patient status. The Brice and McKissock scale (Table 4) classifies four stages of motor function, from mild incapacity (Stage 1) to complete loss of motor, sensory, and sphincter control (Stage 4). The McCormick scale (Table 5) differentiates five stages of neurological function. Stage 1 indicates normal function or minimal dysesthesia; stage 2, mild motor or sensory function, with functional independence; stage 3, moderate deficit; stage 4, severe deficit with limited function and dependence; paraplegia or quadriplegia qualify as stage 5. In the presented sample, patients were analyzed based on several variables, including age, tumor type (intramedullary vs. extramedullary), IONM usage, pre- and postoperative scores on the Prolo, Brice and McKissock, and McCormick scales, as well as any postoperative neurological deficits.

### 2.3. Statistical Analysis

Categorical variables are shown as absolute and relative frequencies. Continuous variables are shown as mean and standard deviations or the median and 25th and 75th percentiles, considering that normality could not be assumed. Linear regression was applied to quantify the relationship between improvement in the Prolo scale and the use of IONM. The Mann–Whitney test was performed to quantify the relationship between the Brice and McKissock and McCormick scales and the use of IONM. In addition, a model of analysis adjusted by the type of tumor (intramedullary vs. extramedullary) was performed. Linear regression was applied to quantify the relationship between the post-surgical neurological changes and the preoperative neurological state and with the use of IONM. Fisher’s test was used to evaluate the significance of the association between postsurgical neurological deficit and the use of IONM.

## 3. Results

The distribution of variables analyzed in the sample (including age, tumor type, use of IONM during the surgery, pre- and postoperative Prolo scores, pre- and postoperative Brice and McKissock scores, pre- and postoperative McCormick scores, and postoperative neurological deficits) is presented in Table 6 and Table 7.

### 3.1. Prolo Scale

**Figure 2:**
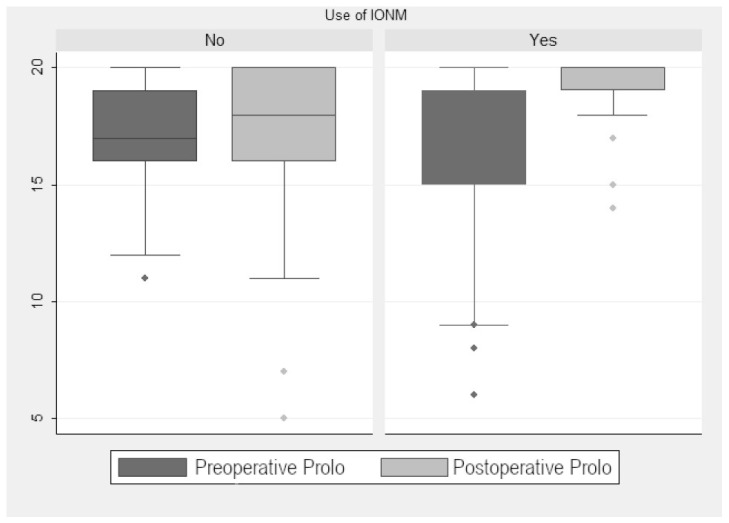
Prolo scale scores.

In the monitored group, the preoperative median Prolo score was 16.33. The postoperative median increased to 19.19 points, so there was a significant improvement (2.95 ± 0.59 points (*p* < 0.001); Figure 1). When we analyzed the improvement according to tumor localization (extramedullary vs. intramedullary), we found a significant postoperative improvement of 2.66 ± 0.81 points (*p* = 0.002). Although non-monitored patients also showed significative improvement, it was less evident (1 ± 0.13 points (*p* < 0.001); Figure 2). The preoperative median in this group was 17 points, with no significant change in the postoperative median (16.9 points). When we compared monitored and non-monitored patients, we found a significant increase of 2.95 ± 0.59 points in the Prolo scale for the monitored group (*p* < 0.001).

### 3.2. Brice and McKissock Scale

**Figure 3:**
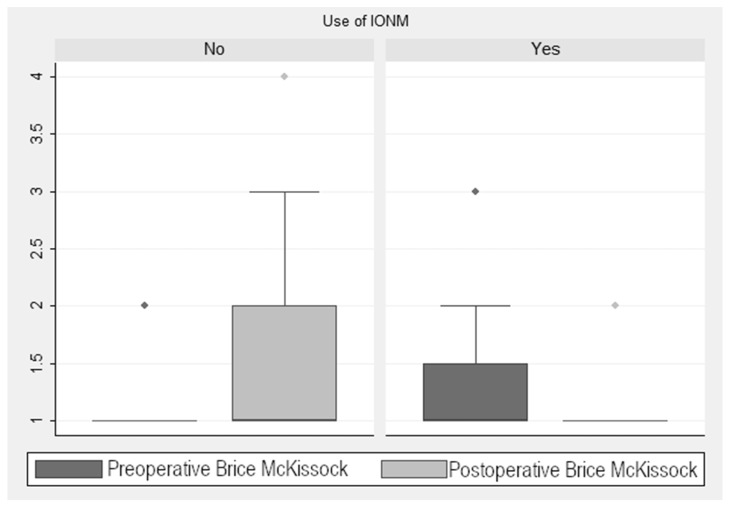
Brice and McKissock Scale scores.

As seen in Figure 3, there was a reduction in the score in the Brice and McKissock scale of 0.58 ± 0.13 points (*p* < 0.001) in the monitored cases. When analyzing this improvement in the monitored cases, taking into account the localization of the tumor (extramedullary vs. intramedullary), we found a decrease of 0.49 ± 0.13 points (*p* < 0.001) in this scale. In the non-monitored patients, there was an increase of 1.6 ± 0.28 points (*p* < 0.001). This finding is relevant, considering the four levels into which this scale is divided and the marked differences in the associated neurological repercussion of each level of the scale (Figure 3). Statistical analysis showed a significant post-pre decrease in the Brice and McKissock scale in the monitored patients compared to the non-monitored ones.

### 3.3. McCormick Scale

**Figure 4:**
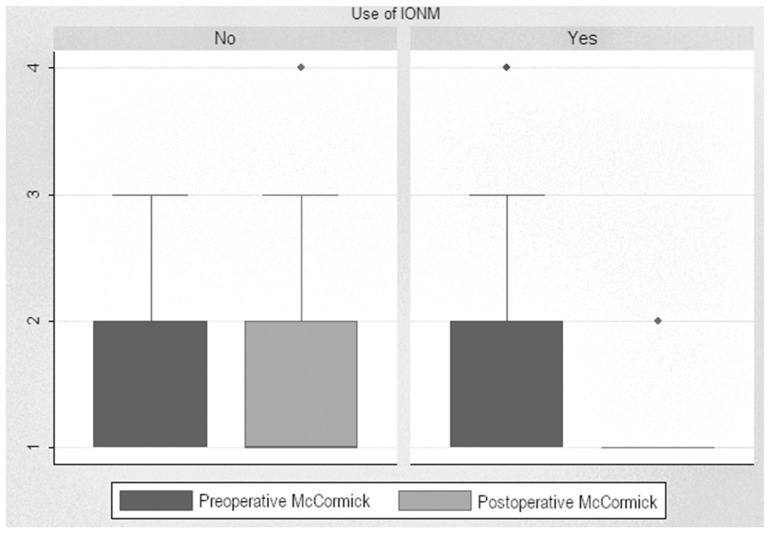
McCormick Scale scores.

Analysis of post-pre scores showed a significant decrease in the scores of monitored patients, with a median decrease from 1.86 to 1.16 (reduction of 0.71 points (*p* < 0.001)). When analyzing this improvement in monitored patients based on tumor localization, we found a decrease of 1.66 points after surgery on intramedullary tumors and a decrease of 0.62 points after surgery on extramedullary tumors. In non-monitored patients, the difference between pre- and postoperative levels was insignificant, even increased, from 1.69 up to 1.78. Statistical analysis showed a significant decrease in the McCormick scale in the monitored patients compared to the non-monitored ones, with a difference of 0.55 points (*p* < 0.001) (Figure 4). 

### 3.4. Clinical Evaluation

We conducted clinical evaluations of the patients between 2 and 3 months postoperatively. Of the 55 patients who had surgery without IONM, 8 experienced new neurological deficits or worsening of pre-existing deficits. These patients include four cases of worsened paraparesis, one of worsened monoparesis, one of worsened tetraparesis, one case of tetraplegia, and one case of worsened sensory deficit. In contrast, only 3 of the 36 patients who underwent a surgery with IONM presented new or worsened neurological deficits (2 of them had worsened paraparesis and 1 patient had loss of urinary sphincter function). These three patients had IONM alerts during the surgery, so IONM predicted the postoperative deficit. Additionally, there were six cases in which there was an IONM event, that is, a warning to the surgeon secondary to a significant neurophysiological change, which was resolved intraoperatively, without any new neurological deficits after the surgery. These data demonstrate that the incidence of new neurological deficits was higher in the non-monitored group (14.5%) compared to the monitored group (8.3%).

## 4. Discussion

The main purpose of our study is to highlight the significance of IONM in surgeries for intradural spinal tumors. The results emphasize its role in assessing the integrity of nervous system structures, detecting intraoperative spinal cord injuries and aiding surgeons in preventing potential neurological damage, if possible. Also, IONM is valuable for predicting postoperative neurological outcomes. In our study, we investigate its impact on postoperative neurological complications considering the anatomical location of the tumor (intramedullary or extramedullary).

The surgical removal of intramedullary spinal cord tumors still carries a significant risk of spinal cord injury and postoperative neurological deficits despite all the recent advances in IONM, microsurgical techniques and neuroimaging [4]. Therefore, incorporating IONM should be standard practice. In the case of intradural extramedullary tumors, where total resection is the goal, there remains a risk of neural pathway injury, making IONM a crucial tool for preventing intraoperative spinal cord damage [12].

The prognosis and outcomes of neuro-oncological interventions largely depend on the possibility of the total resection of the tumor [1,13]. For intramedullary tumors, a transient postoperative motor deficit—predictable through IONM using MEPs, SSEPs, and D-waves—can be justified if it facilitates complete tumor removal, especially in malignant cases [13,14,15]. Conversely, for intradural extramedullary tumors, total resection is expected, and any postoperative deficit is unacceptable [12]. Multimodal IONM in patients at risk of spinal cord injury during surgery has shown sensitivity and specificity rates up to 100% in detecting potential postoperative neurological deficits [16,17,18,19,20,21,22].

Our study demonstrates the significance of IONM in intradural tumor surgeries, revealing an average improvement of 0.58 points in the Brice and McKissock clinical scales for monitored patients, compared to a decline of 1.6 points in unmonitored patients. Similarly, the McCormick scale showed a 0.71-point improvement in monitored patients versus a 0.55-point decline in unmonitored patients. The Prolo scale indicated an average improvement of 2.95 points for monitored patients, contrasted with a 1-point improvement in unmonitored patients, likely reflecting the intrinsic benefits of tumor excision itself, as the Prolo scale also assesses pain and other factors and not only the functional state. These findings may skew the perceived importance of IONM due to the higher likelihood of monitoring intramedullary tumors, which present greater risks. Thus, the prognostic benefits of IONM could potentially be even greater than what our study indicates.

Sala et al. investigated the impact of IONM on intramedullary tumors by comparing the postoperative neurological status of 100 patients, half of whom had their surgery guided by IONM and the remainder from a historical cohort without IONM. They concluded that, at three months post-surgery, patients who had surgery with IONM exhibited superior motor functional outcomes compared to those without [13]. They also noted lasting neurological benefits for monitored patients over a follow-up period of 1 to 1.5 years, particularly among those with mild preoperative neurological deficits (McCormick grades 1 or 2). In our study, the analysis of improvements in monitored patients based on tumor location revealed a small decrease (< 0.5 points) in the Prolo scale for intramedullary tumors compared to extramedullary tumors, likely due to the higher risks associated with intramedullary anatomical location. We observed a decline of 0.49 points in the Brice and McKissock scale and 0.33 points in the McCormick scale for patients with intramedullary tumors, contrasting with overall improvements of 0.58 and 0.71 points for monitored patients, respectively. These results may reflect the greater invasiveness and surgical complexity associated with intramedullary tumors, including increased risk to nervous structures from surgical manipulation and unresected tissue extension. A larger number of negative events are observed during the surgery of intramedullary tumors, making the use of IONM particularly important [21,23,24,25,26,27,28].

While postoperative neurological complication rates are generally lower in extramedullary tumor surgeries compared to intramedullary procedures [12], the intervention for extramedullary intradural tumors is particularly important due to their proximity to the spinal cord. Monitored patients have shown up to a 17.7% benefit in previous studies [29,30], attributed to intraoperative adjustments based on real-time neurophysiological data, which can predict favorable outcomes in approximately 90% of cases. Our study corroborates this finding, demonstrating a lower rate of additional postoperative neurological deficits in patients who underwent IONM compared to those who did not.

Another critical aspect supporting the benefits of IONM in intradural tumor surgeries is the rate of postoperative neurological deficits. In monitored cases, only 8.3% of patients (3 out of 36) experienced worsening neurological status post-surgery, and each of these cases had an IONM event that predicted the outcome. These data are in line with the data reported by Velayutham [21], with the rate of 7.6%. In an additional six cases, IONM events were detected, allowing the surgeon to implement corrective measures. In contrast, 14.5% of non-monitored patients (8 out of 55) experienced severe worsening of their neurological deficits, suggesting that IONM could significantly aid neurosurgeons in making decisions regarding high-risk surgical maneuvers to prevent intraoperative damage.

In this study, we assessed various parameters related to both the pre- and postoperative neurological status of patients using the Prolo, Brice and McKissock, and McCormick scales, and we also tracked the rate of postoperative neurological deficits. We observed functional neurological improvement and a reduction in postoperative deficits among patients with intradural tumors who underwent surgery with IONM support. However, we noted a slight decline in cases involving intramedullary tumors, likely due to the complexities and challenges associated with their resection, as well as the increased risk of a greater spinal cord involvement.

Supporters and skeptics of intraoperative neuromonitoring (IONM) now extend beyond physicians; patients are increasingly aware of this technique and may actively request it during the shared decision-making process. In the context of actual health care systems, it is particularly valuable to gather data on both neurological outcomes and quality of life over an extended follow-up period (years), and it may be a topic for future investigations [31,32].

On the other hand, we consider that our study has some limitations. Due to ethical concerns regarding withholding a potentially beneficial treatment, we were unable to conduct a randomized controlled trial (i.e., Class I evidence with a control group). Consequently, the most reliable data available stem from retrospective studies, which often compare to historical cohorts prior to the adoption of IONM techniques [13,33]. It is important to note that advances in neurosurgical techniques have paralleled the development of IONM methods. To mitigate biases in our results, we employed a “pseudo randomization” approach, designating the “control” group as patients for whom the lead neurosurgeon deemed IONM unnecessary. Given the limited Class I evidence supporting IONM in intradural tumors, its use remains optional in such procedures. Nevertheless, IONM is increasingly recognized for its role in enhancing neurosurgical strategies by providing real-time information about spinal cord functional status—particularly in situations where MEPs are transiently lost or where significant deterioration in D-waves occurs [34].

In this study, we demonstrated clinical improvement and a lower rate of neurological deficits among patients who underwent surgery with IONM support; only 8.3% of monitored patients experienced postoperative deficits, compared to 14.5% in non-monitored patients. Previous research has employed various analytical methods to validate the reliability of intraoperative neurophysiological techniques in predicting the functional integrity of corticospinal tracts during surgery, contributing to increased patient survival. However, these predictions have sometimes come at the cost of long-term recoverable paresis [12,13,18,26,34,35,36,37,38].

Additionally, some authors remark on the significant heterogeneity in the methodologies employed across the trials included in recent meta-analyses. Specifically, there were variations in the monitoring techniques utilized, the warning criteria established, the actions taken in response to warning signals, the reporting of resection extent based on IONM findings, the outcome measures used, and the timing of outcome assessments. These data underscore the necessity for clearly defined and standardized protocols when evaluating these patients, and they emphasize the importance of conducting prospective studies with consistent alarm criteria and outcome measures [31,39].

## 5. Conclusions

Considering the results obtained in the current study, IONM plays a crucial role in the early detection of potential neurological damage during intradural tumor surgeries—encompassing positioning, approach, tumor removal, and spinal reconstruction. It helps decrease the incidence of new neurological deficits and leads to better postoperative functional outcomes. In this study, we demonstrated a reduction in neurological deficits among patients operated with IONM support: only 8.3% of monitored patients experienced postoperative deficits compared to 14.5% in non-monitored. Even though our results match the published data, more studies are needed to obtain sufficient scientific evidence to indisputably support the use of IONM in spinal cord tumors.

## Figures and Tables

**Table 1:** Clinical data of non-monitored patients. OP: operative; BMK: Brice and McKissock; MC: McCormick; P: Prolo.

N°	Gender	Age	PreOP BMK	PostOP BMK	PreOP MC	PostOP MC	PreOP P	PostOP P	PostOP Deficit	Tumor Location	Type of Tumor	Medullar Level
**1**	F	52	2	4	3	4	12	5	Yes	Intramedullary	Ependymoma	C7
**2**	M	50	1	1	1	1	17	18	No	Intramedullary	Ependymoma	Cauda equina
**3**	F	56	1	1	2	2	15	16	No	Extramedullary	Meningioma	D5
**4**	F	55	1	1	2	2	18	19	No	Extramedullary	Meningioma	D2-D3
**5**	F	47	1	1	1	1	18	19	No	Extramedullary	Meningioma	D10-D11
**6**	F	36	1	1	2	2	19	20	No	Extramedullary	Meningothelial meningioma	D2
**7**	F	56	2	2	3	2	13	15	No	Extramedullary	Meningioma	D5
**8**	F	74	1	1	2	2	15	17	No	Extramedullary	Schwannoma	D12-L1
**9**	M	48	1	1	2	1	12	15	No	Intramedullary	PNET	C1-C5
**10**	F	52	1	2	2	3	16	17	No	Extramedullary	Meningioma	Foramen magnum
**11**	F	22	1	1	1	1	18	20	No	Extramedullary	Clear cell meningioma	L1-L2
**12**	F	71	1	2	2	3	14	16	No	Extramedullary	Psammomatous meningioma	C2
**13**	M	20	2	3	3	4	15	11	Yes	Intramedullary	Cavernous angioma	Medullary cone
**14**	M	39	1	1	2	1	17	18	No	Intramedullary	Swchannoma	L5-S1
**15**	M	46	1	1	1	1	20	20	No	Extramedullary	Ependymoma	D12-L1
**16**	F	53	2	1	3	1	18	19	No	Extramedullary	Neurofibroma	C7
**17**	F	20	1	1	1	1	20	20	No	Intramedullary	Hemangioblastoma	C2-C7
**18**	M	27	1	3	1	4	17	11	Yes	Intramedullary	Pilocytic astrocytoma	D4-D7
**19**	M	54	1	1	2	1	15	17	No	Extramedullary	Schwannoma	D11
**20**	F	36	1	1	2	2	16	17	No	Extramedullary	Psammomatous meningioma	D7-D8
**21**	M	60	1	1	2	1	18	19	No	Extramedullary	Schwannoma	L1
**22**	F	61	1	3	2	3	18	13	Yes	Extramedullary	Schwannoma	L4-L5
**23**	F	68	1	1	1	1	19	20	No	Extramedullary	Meningioma	D9
**24**	M	57	1	1	1	1	20	18	No	Extramedullary	Neurinoma	C2
**25**	F	79	1	1	2	1	17	18	No	Extramedullary	Neuroendocrine tumor	D12
**26**	F	26	1	1	1	1	20	20	No	Extramedullary	Neurinoma	L3
**27**	M	76	1	1	2	2	20	20	No	Extramedullary	Neurinoma	L1-L4
**28**	M	34	1	1	1	1	20	20	No	Extramedullary	Neurinoma	L2
**29**	F	48	1	1	1	1	20	20	No	Extramedullary	Neurinoma	L3
**30**	F	61	1	1	2	1	17	18	No	Extramedullary	Meningioma	C1
**31**	M	51	1	1	2	2	16	17	No	Extramedullary	Meningioma	C2-C3
**32**	M	32	1	1	1	1	19	20	No	Extramedullary	Neurofibroma	C1-C2
**33**	M	75	1	1	1	1	20	20	No	Extramedullary	Neurinoma	L1-L2
**34**	F	62	1	1	1	1	20	20	No	Extramedullary	Meningioma	D5-D6
**35**	F	63	1	1	1	1	20	20	No	Extramedullary	Meningioma	D7-D8
**36**	F	83	1	1	2	1	18	19	No	Extramedullary	Neurinoma	D2-D3
**37**	F	58	1	1	1	1	20	20	No	Extramedullary	Meningioma	D3-D4
**38**	F	76	1	1	2	2	17	18	No	Extramedullary	Osseous Metaplastic meningioma	D3
**39**	F	46	1	2	3	2	14	16	No	Extramedullary	Meningioma	D8
**40**	F	51	1	1	2	2	16	17	No	Extramedullary	Meningioma	D5-D9
**41**	F	22	1	3	2	4	18	12	Yes	Intramedullary	Glioblastoma	C3
**42**	M	76	1	2	2	2	13	15	No	Extramedullary	Osseous Metaplastic meningioma	D3
**43**	M	78	1	1	1	1	19	20	No	Extramedullary	Schwannoma	C2-C3
**44**	M	74	1	1	1	1	20	20	No	Extramedullary	Schwannoma	D11
**45**	M	35	1	3	1	4	19	13	Yes	Extramedullary	Fibrolipoma	D1-D3
**46**	F	75	2	2	3	2	11	14	No	Extramedullary	Neurinoma	D5-D6
**47**	F	78	1	1	2	1	17	19	No	Extramedullary	Meningioma	D5
**48**	F	71	1	1	1	1	19	19	No	Extramedullary	Meningioma	C2
**49**	F	56	1	1	1	1	20	20	No	Extramedullary	Psammomatous meningioma	D4
**50**	F	69	1	1	1	2	17	15	No	Extramedullary	Meningioma	D4-D5
**51**	F	42	1	1	2	2	16	16	No	Extramedullary	Psammomatous meningioma	D3-D5
**52**	M	59	1	1	1	1	16	17	No	Extramedullary	Arachnoid cyst	L1-L2
**53**	M	67	2	4	3	4	12	7	Yes	Extramedullary	Psammomatous meningioma	D10
**54**	F	53	1	1	2	2	16	17	No	Extramedullary	Metastatic breast cancer	L1
**55**	F	61	2	4	2	4	13	7	Yes	Extramedullary	Ependymoma	C6

**Table 2:** Clinical data of monitored patients. OP: operative; BMK: Brice and McKissock; MC: McCormick; P: Prolo.

N°	Gender	Age	PreOP BMK	PostOP BMK	PreOP MC	PostOP MC	PreOP P	PostOP P	IONM Event	PostOP Deficit	Tumor Location	Type of Tumor	Medullar Level
**1**	M	46	1	1	1	1	9	20	No	No	Extramedullary	Schwannoma	D12-L1
**2**	M	44	1	1	2	1	17	19	No	No	Extramedullary	Neurinoma	L2
**3**	F	48	3	1	4	2	8	17	No	No	Extramedullary	Myxopapillary ependymoma	L5-S1
**4**	F	43	1	1	2	1	17	20	No	No	Intramedullary	Ependymoma	L3-L5
**5**	F	48	1	1	2	1	18	20	No	No	Extramedullary	Neurinoma	L3
**6**	M	23	1	1	1	1	20	20	No	No	Extramedullary	Neurinoma	C4-C5
**7**	F	48	2	1	3	1	13	19	No	No	Extramedullary	Neurinoma	L3
**8**	M	50	1	1	1	1	19	20	No	No	Extramedullary	Myxopapillary ependymoma	L1-L2
**9**	M	39	2	1	2	1	17	20	No	Yes	Extramedullary	Ependymoma	D10-D12
**10**	M	40	2	1	3	1	15	19	No	Yes	Extramedullary	Hemangioblastoma	D4-D7
**11**	M	26	1	2	1	2	20	15	Yes	Yes	Intramedullary	Fibrolipoma	D1-D3
**12**	F	59	1	1	1	1	19	20	No	No	Extramedullary	Paraganglioma	L4
**13**	M	71	2	1	3	1	14	20	Yes	No	Extramedullary	Meningioma	D3
**14**	M	39	1	1	1	1	20	20	No	No	Extramedullary	Schwannoma	L5-S1
**15**	M	41	1	1	2	1	18	20	No	No	Extramedullary	Neurinoma	L1
**16**	F	75	1	1	2	1	17	20	No	No	Extramedullary	Meningioma	D5-D6
**17**	M	58	1	1	1	1	20	20	No	No	Extramedullary	Schwannoma	D7
**18**	M	33	1	1	1	1	19	20	No	No	Intramedullary	Schwannoma	L2-L3
**19**	M	32	1	1	1	1	18	20	No	No	Extramedullary	Neurinoma	C6-C7
**20**	M	66	2	1	3	2	12	17	Yes	No	Extramedullary	Angiomatous meningioma	D4
**21**	M	68	1	1	1	1	19	20	No	No	Extramedullary	Meningioma	C1-C2
**22**	F	79	1	1	2	1	17	20	No	No	Extramedullary	Psammomatous meningioma	D9
**23**	F	34	1	1	2	1	17	19	Yes	No	Extramedullary	Neurinoma	D9-D10
**24**	M	75	2	1	4	2	11	18	No	No	Extramedullary	Schwannoma	L2
**25**	M	52	3	1	4	2	8	17	No	No	Extramedullary	Dural fistula	D10
**26**	F	62	1	1	2	1	15	19	No	No	Extramedullary	Schwannoma	L1
**27**	M	49	1	1	1	1	18	20	No	No	Extramedullary	Paraganglioma	L1-L2
**28**	F	78	1	1	1	1	19	20	Yes	No	Extramedullary	Meningioma	D5
**29**	F	22	1	1	1	1	20	20	No	No	Extramedullary	Ependymoma	C3-D1
**30**	M	41	1	1	1	1	18	20	No	No	Extramedullary	Neurinoma	L3-L5
**31**	F	37	3	2	4	2	6	14	No	No	Extramedullary	Fusocellular sarcoma	L2-L3
**32**	F	76	1	1	2	1	16	19	No	No	Extramedullary	Psammomatous meningioma	D8-D9
**33**	M	57	1	1	1	1	18	20	Yes	No	Intramedullary	Ependymoma	D11-D12
**34**	M	81	1	1	2	1	16	19	Yes	No	Extramedullary	Paranganglioma	D3-D4
**35**	F	64	1	1	1	1	20	20	No	No	Extramedullary	Filum terminale cyst	D10-D12
**36**	M	20	1	1	1	1	20	20	No	No	Extramedullary	Cavernous angioma	Medullary cone

**Table 3:** Prolo scale. Overall rating: sum of scores from the four evaluated areas. Scores of 4–8: poor QoL; 9–12: fair QoL; 13–16: good QoL; 17–20: excellent QoL. QoL: Quality of life. NSAIDs: Non-steroid anti-inflammatory drugs [6,7].

Prolo Scale				
	Pain	Function	Economic	Medication
1	Unbearable	Total incapacity	Unable to do tasks around home	Parenteral strong opioids
2	Severe	Can do activities at home	Able to do tasks around home but unable to perform paid work	Regular use of parenteral strong opioids
3	Moderate	Activities outside home w/limitation	Able to work at sedentary capacity	Regular use of parenteral weak opioids
4	Mild	Limitation w/strenuous activities	Able to work at moderate capacity	Regular use of NSAIDs or occasional opioids
5	None	Able to do everything	Able to work at heavy capacity or previous job	None or occasional NSAIDs

**Table 4:** Brice and McKissock scale. The rating depends on the neurological clinical situation of the patient as indicated above [8,9].

Brice and McKissock Scale	
Grade	Severity of the Neurological Lesion
1	Mild (able to walk)
2	Moderate (able to move legs, but not antigravity)
3	Severe (slight residual motor and sensory function)
4	Complete (no motor, sensory or sphincter function below level of lesion

**Table 5:** McCormick Scale [11].

McCormick Scale	
I	Intact neurologically, normal ambulation, minimal dysesthesia
II	Mild motor or sensory deficit, functional independence
III	Moderate deficit, limitation of function, independent with external aid
IV	Severe motor or sensory deficit, limited function, dependent
V	Paraplegia or quadriplegia, even with flickering movement

**Table 6:** Distribution of variables in the two cohorts (IONM vs. Non- IONM): Mean and standard deviation of the variables, as well as the absolute and relative frequency of type of tumor and postoperative neurological deficit.

	Without IONM	With IONM
N (patients)	36	55
Age (years) mean ± SD	50.66 ± 17.48	54.49 ±17.06
Preoperative Prolo mean ± SD	16.33 ± 3.83	17.09 ± 2.54
Postoperative Prolo mean ± SD	19.19 ± 1.47	16.98 ± 3.57
Preoperative Brice McKissock (mean ± SD)	1.33 ± 0.63	1.12 ± 0.33
Postoperative Brice McKissock (mean ± SD)	1.05 ± 0.23	1.45 ± 0.87
Intramedullary tumors (observed freq., relative freq.)	4 (0.11, 11%)	8 (0.14, 14%)
Extramedullary tumors (observed freq., relative freq.)	32 (0.88, 88%)	47 (0.85, 85%)
Postoperative Neurological Deficits (observed freq., relative freq.)	8 (0.22, 22%)	3 (0.05, 5%)

**Table 7:** Mean differences in the pre- and postoperative scores in Prolo, Brice and McKissock and McCormick scales.

	Differences (Mean)	*p* (Statistical Significance)
Post-Pre Prolo score in patients with IONM	2.95	0.002
Post-Pre Prolo score in patients without IONM	1	<0.001
Post-Pre Brice and McKissock in patients with IONM	−0.58	<0.001
Post-Pre Brice and McKissock in patients without IONM	1.6	<0.001
Post-Pre McCormick in patients with IONM	−0.71	<0.001
Post-Pre McCormick in patients without IONM	0.55	<0.001

## Data Availability

The original contributions presented in this study are included in the article. Further inquiries can be directed to the corresponding author(s).

## References

[1] Duong L.M., McCarthy B.J., McLendon R.E., Dolecek T.A., Kruchko C., Douglas L.L., Ajani U.A. (2012). Descriptive epidemiology of malignant and nonmalignant primary spinal cord, spinal meninges, and cauda equina tumors, United States, 2004–2007. Cancer.

[2] Weber C., Gulati S., Jakola A.S., Habiba S., Nygaard Ø.P., Johannesen T.B., Solheim O. (2014). Incidence rates and surgery of primary intraspinal tumors in the era of modern neuroimaging: A national population-based study. Spine.

[3] Singh P.R., Pandey T.K., Sharma R.K., Ahmad F., Kumar A., Agarwal A. (2021). Tumor Occupancy Ratio-An Imaging Characteristic Prognosticating the Surgical Outcome of Benign Intradural Extramedullary Spinal Cord Tumors. Int. J. Spine Surg..

[4] Sala F., Bricolo A., Faccioli F., Lanteri P., Gerosa M. (2007). Surgery for intramedullary spinal cord tumors: The role of intraoperative (neurophysiological) monitoring. Eur. Spine J..

[5] Sala F. (2010). Intraoperative neurophysiology is here to stay. Childs. Nerv. Syst..

[6] Prolo D.J., Oklund S.A., Butcher M. (1986). Toward uniformity in evaluating results of lumbar spine operations. A paradigm applied to posterior lumbar interbody fusions. Spine.

[7] Vanti C., Prosperi D., Boschi M. (2013). The Prolo Scale: History, evolution and psychometric properties. J. Orthop. Traumatol..

[8] Brice J., Mckissock W. (1965). Surgical treatment of malignant extradural spinal tumours. Br. Med. J..

[9] Bellut D., Burkhardt J.-K., Mannion A.F., Porchet F. (2015). Assessment of outcome in patients undergoing surgery for intradural spinal tumor using the multidimensional patient-rated Core Outcome Measures Index and the modified McCormick Scale. Neurosurg. Focus.

[10] McCormick P.C., Stein B.M. (1990). Intramedullary tumors in adults. Neurosurg. Clin. N. Am..

[11] McCormick P.C., Torres R., Post K.D., Stein B.M. (1990). Intramedullary ependymoma of the spinal cord. J. Neurosurg..

[12] Ghadirpour R., Nasi D., Iaccarino C., Giraldi D., Sabadini R., Motti L., Sala F., Servadei F. (2015). Intraoperative neurophysiological monitoring for intradural extramedullary tumors: Why not?. Clin. Neurol. Neurosurg..

[13] Sala F., Palandri G., Basso E., Lanteri P., Deletis V., Faccioli F., Bricolo A. (2006). Motor evoked potential monitoring improves outcome after surgery for intramedullary spinal cord tumors: A historical control study. Neurosurgery.

[14] Constantini S., Miller D.C., Allen J.C., Rorke L.B., Freed D., Epstein F.J. (2000). Radical excision of intramedullary spinal cord tumors: Surgical morbidity and long-term follow-up evaluation in 164 children and young adults. J. Neurosurg..

[15] Jallo G.I., Freed D., Epstein F. (2003). Intramedullary spinal cord tumors in children. Child’s Nerv. Syst..

[16] Sutter M., Eggspuehler A., Grob D., Jeszenszky D., Benini A., Porchet F., Mueller A., Dvorak J. (2007). The validity of multimodal intraoperative monitoring (MIOM) in surgery of 109 spine and spinal cord tumors. Eur. Spine J..

[17] Sutter M., Eggspuehler A., Muller A., Dvorak J. (2007). Multimodal intraoperative monitoring: An overview and proposal of methodology based on 1,017 cases. Eur. Spine J..

[18] Nuwer M.R., Packwood J.W. (2008). Monitoring during the surgical treatment of scoliosis. Intraoperative Monitoring of Neural Function.

[19] Fehlings M.G., Brodke D.S., Norvell D.C., Dettori J.R. (2010). The evidence for intraoperative neurophysiological monitoring in spine surgery: Does it make a difference?. Spine.

[20] Scibilia A., Terranova C., Rizzo V., Raffa G., Morelli A., Esposito F., Mallamace R., Buda G., Conti A., Quartarone A. (2016). Intraoperative neurophysiological mapping and monitoring in spinal tumor surgery: Sirens or indispensable tools?. Neurosurg. Focus.

[21] Velayutham P., Rajshekhar V., Chacko A.G., Krothapalli Babu S. (2016). Influence of tumor location and other variables on predictive value of intraoperative myogenic motor-evoked potentials in spinal cord tumor surgery. World Neurosurg..

[22] Tropeano M.P., Rossini Z., Franzini A., Capo G., Olei S., De Robertis M., Milani D., Fornari M., Pessina F. (2024). Multimodal Intraoperative Neurophysiological Monitoring in Intramedullary Spinal Cord Tumors: A 10-Year Single Center Experience. Cancers.

[23] Wiedemayer H., Fauser B., Sandalcioglu I.E., Schäfer H., Stolke D. (2002). The impact of neurophysiological intraoperative monitoring on surgical decisions: A critical analysis of 423 cases. J. Neurosurg..

[24] Kothbauer K.F. (2007). Intraoperative neurophysiologic monitoring for intramedullary spinal-cord tumor surgery. Clin. Neurophysiol..

[25] Ando M., Tamaki T., Yoshida M., Kawakami M., Kubota S., Nakagawa Y., Iwasaki H., Tsutsui S., Yamada H. (2015). Intraoperative spinal cord monitoring using combined motor and sensory evoked potentials recorded from the spinal cord during surgery for intramedullary spinal cord tumor. Clin. Neurol. Neurosurg..

[26] Jin S.-H., Chung C.K., Kim C.H., Choi Y.D., Kwak G., Kim B.E. (2015). Multimodal intraoperative monitoring during intramedullary spinal cord tumor surgery. Acta Neurochir..

[27] Tanaka S., Hirao J., Oka H., Akimoto J., Takanashi J., Yamada J. (2015). Intraoperative monitoring during decompression of the spinal cord and spinal nerves using transcranial motor-evoked potentials: The law of twenty percent. J. Clin. Neurosci..

[28] Kim D.G., Son Y.R., Park Y.S., Hyun S.J., Kim K.J., Jahng T.A., Kim H.J., Park K.S. (2016). Differences in Multimodality Intraoperative Neurophysiological Monitoring Changes Between Spinal Intramedullary Ependymoma and Hemangioblastoma. J. Clin. Neurophysiol. Off. Publ. Am. Electroencephalogr. Soc..

[29] Forster M.-T., Marquardt G., Seifert V., Szelényi A. (2012). Spinal cord tumor surgery—Importance of continuous intraoperative neurophysiological monitoring after tumor resection. Spine.

[30] Costa P., Peretta P., Faccani G. (2013). Relevance of intraoperative D wave in spine and spinal cord surgeries. Eur. Spine. J..

[31] Liu K., Ma C., Li D., Li H., Dong X., Liu B., Yu Y., Fan Y., Song H. (2023). The role of intraoperative neurophysiological monitoring in intramedullary spinal cord tumor surgery. Chin. Neurosurg. J..

[32] Siller S. (2024). Multimodal intraoperative neurophysiological monitoring may better predict postoperative distal upper extremities’ complex-functional outcome than spinal and muscular motor evoked potentials alone in high-cervical intramedullary spinal cord tumor surgery. Clin. Neurophysiol..

[33] Asazuma T., Toyama Y., Suzuki N., Fujimura Y., Hirabayshi K. (1999). Ependymomas of the spinal cord and cauda equina: An analysis of 26 cases and a review of the literature. Spinal Cord..

[34] Deletis V., Sala F. (2008). Intraoperative neurophysiological monitoring of the spinal cord during spinal cord and spine surgery: A review focus on the corticospinal tracts. Clin. Neurophysiol..

[35] Deletis V. (2002). Intraoperative Neurophysiology and Methodologies Used to Monitor the Functional Integrity of the Motor System. Neurophysiology in Neurosurgery.

[36] Kothbauer K.F., Deletis V., Shils J. (2002). Motor Evoked Potential Monitoring for Intramedullary Spinal Cord Tumor Surgery. Neurophysiology in Neurosurgery.

[37] MacDonald D.B., Dong C.C.J. (2008). Spinal cord monitoring during descending aortic procedures. Intraoperative Monitoring of Neural Function.

[38] Avila E.K., Elder J.B., Singh P., Chen X., Bilsky M.H. (2013). Intraoperative neurophysiological monitoring and neurologic outcomes in patients with epidural spine tumors. Clin. Neurol. Neurosurg..

[39] Azad T.D., Pendharkar A.V., Nguyen V., Pan J., Connolly I.D., Veeravagu A., Popat R., Ratliff J.K., Grant G.A. (2018). Diagnostic utility of intraoperative neurophysiological monitoring for intramedullary spinal cord tumors: Systematic review and meta-analysis. Clin. Spine Surg..

